# Real-world evaluation of an acceptance and commitment therapy–based group programme for breast cancer survivors with fear of cancer recurrence

**DOI:** 10.1007/s00520-023-08179-3

**Published:** 2023-11-15

**Authors:** Fiona Sinclair, David Gillanders, Natalie Rooney, Christine Bonathan, Kirsty Hendry, Philip McLoone, Christopher Hewitt

**Affiliations:** 1 NHS Greater Glasgow & Clyde, Glasgow, Scotland; 2 School of Health in Social Sciences, University of Edinburgh, Edinburgh, Scotland; 3 Maggie’s Forth Valley, Larbert, Scotland; 4 School of Health & Wellbeing, University of Glasgow, Glasgow, Scotland; 5 NHS Lothian, Edinburgh, Scotland

**Keywords:** Breast cancer, Fear of cancer recurrence, Acceptance and commitment therapy, Patient support, Group support

## Abstract

**Purpose:**

To evaluate the effectiveness and acceptability of a 6-week acceptance and commitment therapy (ACT)–based group programme on participants’ fear of cancer recurrence (FCR), quality of life (QoL), psychological distress and psychological flexibility at the end of the programme and 12-week follow-up.

**Methods:**

A one-group, post-test service evaluation of a real-world psychological programme was carried out to evaluate collected outcome measures and attendance for a total of 21 groups facilitated between 2017 and 2019. Participants were breast cancer survivors who attended a 6-week group programme led by NHS clinicians. Descriptive statistics and repeated measures ANOVA analyses were carried out for each outcome measure. Attendance levels were examined to assess acceptability.

**Results:**

A total of 97 group participants who had completed curative treatment for breast cancer took part. Of whom, 89% completed at least 4 of the 6 weekly group sessions and 76% attended the 12-week follow-up session. Eighty-four (87%) participants returned outcome measures at all three time points relative to group participation (T1 = pre, T2 = post T3 = 12-week follow-up). Group participants were female, mean age 51.9 years. FCR was highest at T1 (mean 25.2, SD 4.7), reduced T2 (mean 21.2, SD 5.4) and further lowered T3 (mean 19.5, SD 6.2). This difference was statistically significant (*p* < 0.001). QoL was lowest at T1 (mean 62.4, SD 15.7), increased T2 (mean 71.7, SD 18.1) and further increased at T3 (mean 75.9, SD 17.5). This difference was statistically significant (*p* < 0.001). Psychological distress measures were shown to reduce, and psychological flexibility increased.

**Conclusions:**

This real-world evaluation of an ACT-based group programme led to improvements in FCR, QoL, psychological distress and psychological flexibility in this population. This evaluation provides basis for further investigation to determine if these results can be replicated by controlled research design across diverse populations.

## Introduction

It is common and normal for people to experience a fear of cancer coming back following curative treatment. Breast cancer recurrence usually occurs within the first 10 years following curative treatment, with slightly over 50% of women from a large sample found to be cancer free at 10-year follow-up, and evidence of recurrence in a small number of women found up to 32 years following initial diagnosis [1]. For many breast cancer survivors, thoughts of the possibility of cancer recurrence are manageable and this may have a positive impact in increasing motivation to attend follow-up appointments, adhere to maintenance treatment regimens and lead a healthy lifestyle [2]. However, for some, fear of cancer recurrence (FCR) is debilitating [3]. Individuals with elevated levels of FCR over longer periods of time have been found to have reduced quality of life with increased emotional distress, isolation, hyper-vigilance of physical symptoms, fatigue, and cognitive issues [4, 5]. Furthermore, it has been shown that FCR persists over time, sometimes for many years following active treatment, indicating need for intervention [4]. Meta-analysis has revealed that overall women exhibit higher FCR than men [6]. Furthermore, FCR is a significant problem for breast cancer survivors and can persist many years after completion of active treatment [2].

However, it is still not commonplace for those with FCR to be able to access psychological support [7]. There is currently no consensus on the best supportive care approach for someone with elevated FCR. In efforts to address this, a 2-day colloquium was held at the University of Ottawa during which, experts in the field met with the aim of reaching agreement regarding the diagnostic clinical characteristics of FCR [8]. Five key diagnostic criteria were identified following three rounds of discussion: problematic thoughts, maladaptive coping, functional impairment, high levels of distress and barriers to future planning.

Rates of death from cancer continue to decline [9] and survival rates continue to rise in the first year [10] and 5 years [11] following diagnosis, with advancements in research and treatment options. However, with this comes an increasing number of people living with the physical and emotional impact following treatment for cancer. A recent systematic review found that 59% of cancer survivors were found to have moderate FCR and a further 19% were found to have severe FCR [12], evidencing a need for effective treatment of FCR as a common challenge faced by people following cancer treatment. Despite this demonstrably prevalent problem, FCR is reported as one of the most commonly unmet needs of patients across cancer types [3].

Systematic review and meta-analysis of psychological interventions for FCR, examining data from 23 psychological interventions including 21 randomised controlled trials (RCTs), found that psychological interventions are effective in treating FCR. Furthermore, contemporary cognitive behavioural therapy (CBT) interventions, which includes acceptance and commitment therapy (ACT), were more effective than traditional CBT interventions [13].

ACT interventions have been found to benefit individuals with a range of chronic health conditions. The overall aim of ACT is to increase psychological flexibility [14]. Psychological flexibility is defined as the ability to be aware of and open to unwanted emotions and sensations, whilst continuing to live according to deeply held personal values. [15].

A pilot RCT examined three different treatments for FCR: a six-session ACT group intervention, survivorship education and enhanced usual care [16]. This trial randomly assigned 91 women who had completed treatment for breast cancer to one of these three arms. The results found that the ACT intervention was able to produce a reduction in measures of FCR severity and increase in QoL, pre, post and follow-up. It was found that participants within the other two arms had minimal change in outcome measures.

Further support for the utility of an ACT-based intervention to address FCR was found within the ConquerFear RCT, which compared the efficacy of a 5-session face-to-face ACT-based intervention to a control intervention based on relaxation training for those with elevated FCR [17]. Participants taking part in this RCT were survivors of melanoma, breast cancer or colorectal cancer. Both interventions were found to have good retention rates: 70% and 67% respectively. Participants who took part in the ACT-based intervention were found to have a significant improvement in anxiety, psychological distress and quality of life scores compared to the control group and a significant reduction in FCR. These benefits were found to be maintained at the 6-month follow-up. Further evaluation revealed that those with higher FCR scores showed greatest benefit from the ACT-based intervention, compared to relaxation training [18]. This intervention has also been piloted in a self-guided, online format, namely iConquerFear, with initial promising results for feasibility and efficacy [19].

The group programme described within this one-group, post-test, real-world service evaluation was developed in 2017 in response to the unmet support needs of people experiencing FCR within a UK NHS cancer treatment setting [3, 8]. The programme was developed by NHS clinicians actively involved in the care of people affected by cancer. An ACT-based model was utilised, based on the promising findings described above.

## Objectives

- To evaluate the benefits of a 6-week ACT-based group programme for patients who have completed treatment for breast cancer.
- To evaluate outcome measure scores 12 weeks following group participation.
- To examine the acceptability of the group programme within this population as measured by uptake and retention.

## Methods

### Group programme participants

Those eligible for the group programme were:

i) Women who had completed curative active treatment for primary breast cancer (stage 0–3). Those prescribed maintenance treatment, such as endocrine therapy, Herceptin or adjuvant bisphosphonates were eligible.
ii) Not demonstrating evidence of recurrence at the point of starting the programme.
iii) Age 18 years or older.
iv) Proficient in English.

Following inception of this group intervention, awareness was raised amongst colleagues, as potential referrers, by the group facilitators who were an NHS clinical psychologist and a specialist therapeutic radiographer. The group facilitators attended multi-disciplinary team meetings within oncology settings across the West of Scotland to present the programme.

Those with cancer or undergoing active treatment as well as patients experiencing complex psychological/psychiatric conditions, substance misuse or suicidal ideation, as evidenced by medical notes, were not deemed to be appropriate for this intervention. In cases where such patients were referred, they were provided with advice on alternative, potentially suitable support available.

### Referral process

Posters and leaflets were produced and distributed within hospital oncology settings, local cancer support charities and GP surgeries. Referrals were made predominantly by hospital-based NHS clinicians, with the patients’ verbal consent, who identified FCR using clinical judgement during routine follow-up appointments. Self-referrals and referrals from third sector charitable organisations, with verbal consent, were also welcomed. Third sector organisations are defined as those providing charitable support independently of this intervention who could signpost or refer directly to this group intervention. All referrals were considered by the facilitators for suitability utilising access to medical records and discussion during weekly referral meetings. Following each referral meeting, facilitators followed up each referral with a review call to discuss the group programme and their expectations of this.

### Service development and delivery

The group programme took place over a period of 3 years from January 2017 to December 2019, with 8 groups run during 2017, 8 groups in 2018 and 5 groups in 2019. The programme format and materials were developed by the group facilitators, and development was further informed by supervision from a clinical psychologist with expertise in ACT supervision and training.

The 6-week programme was designed to encompass the core processes defined. See Table 1 for a breakdown of the themes covered within each session. Following each session, participants received handouts containing a summary of the session and home-based exercises linked to the session content.

**Table 1:** Summary of weekly FCR group programme themes and content

Session	Target ACT core processes	Exercises	Homework
1	Acceptance and contact with the present moment	Ice breaker discussing participant expectations of the group, explanation the aims of the group and basis of ACT, introduction of creative hopelessness through exploration of coping styles and leaves on a stream mindfulness exercise	Read Dr Peter Harvey ‘After treatment finishes – then what?’ article. Spend time getting to know own coping style, listing different things you do, how that impacts anxiety levels and QoL. Practice mindfulness
2	Acceptance and contact with the present moment	Educational session led by therapeutic radiographer. Interactive exploration of the facts and myths relating to lifestyle and coping. Group discussion of the triggers of FCR. Three-minute breathing space mindfulness exercise	Read over value check list in preparation for week 3 session. Practice mindfulness
3	Values and contact with the present moment	Introduction to values and shown goals vs values video by Russ Harris. Earthquake metaphor introduced. Values checklist/cards breakout exercise. Introduced to Values Bullseye. Beach ball and elephant metaphor presented. Mindfulness of the hand exercise	Write down intended actions in line with values. Take time to think about what is important within the four areas of the Bullseye and reflect on how much time you give to them. Practice mindfulness
4	Values, defusion, self-as-context, committed action and present moment awareness	Revisit bullseye and review any actions taken by the group in the past week. Emphasis on the impact our thoughts can have and that we are not our thoughts. Passengers on the bus metaphor illustrated with group participation. Concept of defusion discussed and stepping back from tricky thoughts—Kick your buts, Sushi Train, Thanking your mind. Mindfulness eating exercise	Write down intended action in line with values. Spend time trying out the tools described this week to help with stepping back from thoughts. Practice mindfulness
5	Values, self-as-context, committed action and defusion	Revisit bullseye and review any actions taken by the group in the past week. Bold move exercise. Exploration of barriers to committed action. In-depth discussion of the impact of emotions. Sky metaphor introduced. The importance of self-compassions. Acceptance of emotions mindfulness exercise	Work on intended actions. Try out the bold move card exercise. Re-watch the Russ Harris ‘Struggle switch’ video. Try catching and stepping back from difficult thoughts. Practice mindfulness
6	Acceptance, committed action and present moment awareness	Group introduced to the Choice Point. Recap of the content covered through programme. Emphasis of the importance of noticing progress. Group invited to suggest exercises/topics covered to revisit or go into further detail. Another kind of self-mindfulness exercise	Work on choice point. Read over the course summary. Completes the end of course Blueprint. Read over the information sent regarding other support available. Practice mindfulness

### Data collection

Each group lasted 6 consecutive weeks, 2 hours per week, and all participants had the opportunity to attend an optional session, 12 weeks after participation to discuss their experience following the programme. Sessions were face-to-face within hospital and community-based settings across Greater Glasgow & Clyde and Lanarkshire, with the aim of optimising accessibility within the parameters of the NHS health board. There was a maximum of 15 participants per group and participation was voluntary. There was no participant waiting list following referral, with group scheduling tailored to demand, by the facilitators.

To evaluate whether this group programme was meeting its aims and providing a benefit to the participants, data were accessed and analysed by an experienced PhD-trained researcher who was not involved in the design or delivery of the groups, or the collection of outcome measures. The outcome measures were sent to all participants by post at three time points: prior to starting (T1), after completion (T2) and 12 weeks following group programme participation (T3). Outcome measures were self-administered and returned by post via the provided pre-paid addressed envelope. Demographic data relating to group participants’ age, socio-economic status (based on Scottish Index of Multiple Deprivation calculated from postcode) and ethnicity were also collated.

### Outcome measures

Fear of Cancer Inventory-Short Form (FCRI-SF) [20] — Aims to measure the severity of fears of cancer recurrence. It is a 9-item measure with higher scores indicating higher levels of FCR. This tool has shown excellent internal consistency (*α* = 0.95) [21].

Functional Assessment of Cancer Therapy–Breast (FACT-B) [22] — Provides a measure of health-related quality of life (HRQOL) for people who have had a diagnosis of breast cancer. It is a 37-item measure with higher scores indicating higher quality of life. Total score was analysed within this service evaluation. This tool has been found to have excellent internal consistency (*α* = 0.90) [22].

Comprehensive Assessment of Acceptance and Commitment Therapy Processes (CompACT) [23] — A 23-item tool developed to assess an individual’s psychological flexibility. This tool has been found to have acceptable internal consistence (*α* = 0.79–0.83) [24].

The Patient Health Questionnaire (PHQ-9) [25] — This 9-item questionnaire aims to measure overall severity of symptoms of depression. This tool has shown good internal consistency (*α* = 0.89) [25].

Generalised Anxiety Disorder Assessment (GAD-7) [26] — This 7-item questionnaire was developed as a measure of symptoms of generalised anxiety disorder. This tool has been found to have excellent internal consistency (*α* = 0.92) [26].

## Analysis

Demographic information was examined using descriptive statistics. The data were assessed for assumption of normality using skewness and kurtosis statistics as well as homogeneity of variance. The data were checked for normal distribution at each time point as indicated by boxplot and Shapiro–Wilk (*p* > 0.05). The data were analysed using a one-way repeated measures ANOVA design to assess any changes in the outcome measures across time. Mauchly’s test of sphericity was used to indicate whether the assumption of sphericity was met. A *p*-value < 0.05 was used as the statistical significance level. Significant differences between data collection points were assessed using the planned comparisons procedure within the repeated measures ANOVA. Statistical analysis was carried out using IBM SPSS Statistics version 28.0.

## Results

### Participants

**Fig. 1:**
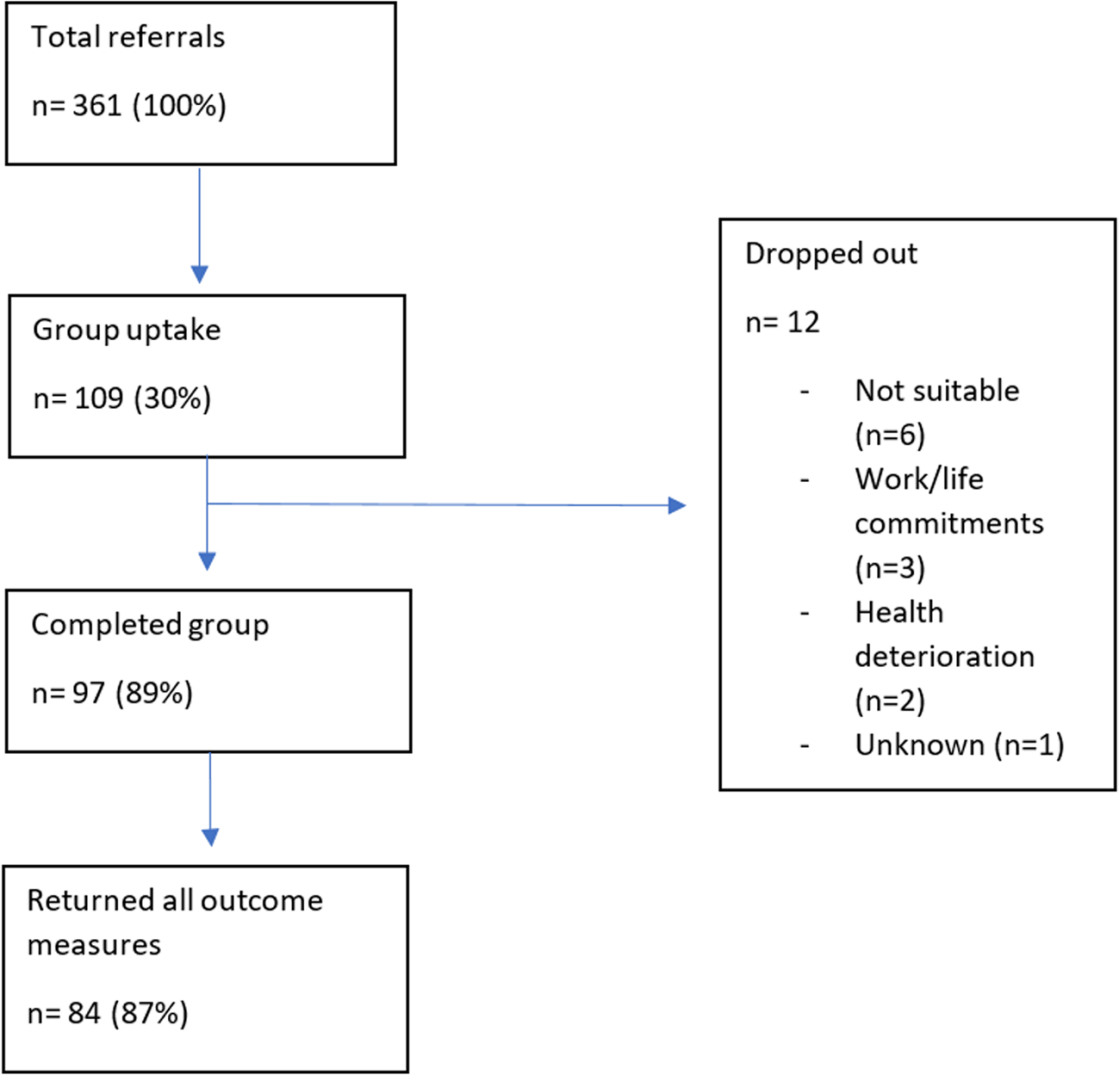
Flow diagram illustrating total referrals, uptake and retention

Between January 2017 and December 2019, 361 patients who had completed active treatment for breast cancer were referred to the FCR group programme. These referrals consisted of 288 (79.8%) referrals from NHS clinicians, 40 (11.1%) referrals from third sector organisations, 30 (8.3%) self-referrals and 3 (0.8%) referrals from general practitioners. See the flow diagram within Fig. 1 which illustrated the total referrals received as well as group programme uptake, completion and outcome measures returned.

All participants were female and had completed treatment with curative intent for breast cancer. Baseline characteristics illustrating age-range, Scottish Index of Multiple Deprivation (SIMD) [27] as a relative measure of deprivation (lower score equals higher deprivation) and ethnicity are shown in Table 2. Furthermore, participants started the programme on average 16.4 months since breast cancer diagnosis and 9.8 months since completion of active treatment.

**Table 2:** Baseline age and SIMD characteristics of evaluated participating sample

	Category	Total sample *n* (%)
Age range	< 40	11 (11)
	40–50	30 (31)
	51–60	39 (40)
	> 61	17 (18)
SIMD	1	12 (12)
	2	25 (26)
	3	15 (15)
	4	19 (20)
	5	26 (27)
Ethnicity	White British	95 (97)
	White Polish	2 (3)
Total sample		97 (100)

### Acceptability

There was an uptake rate of 30.2% (*n* = 109/361). There was an 89% (*n* = 97/109) retention rate based on participants completing at least four out of six sessions. Seventy-six percent (*n* = 74/109) who started the programme also attended the 12-week follow-up session. Eighty-seven percent (*n* = 84/109) returned outcome measures at all three time points.

### Impact

All data met assumptions for parametric analysis. Assumptions for sphericity were also met, except for the GAD-7 measure, to which the Greenhouse–Geisser correction was applied. Table 3 shows the summary of the results, with effect sizes.

**Table 3:** Descriptive statistics and results of mixed ANOVA analysis of FCR outcome measure split by between groups factor of administration and within groups factor of time point

Outcome measure	T1		T2		T3		Time point *F*_(2, 166)_	*η*_p_^2^
	Mean	SD	Mean	SD	Mean	SD		
FCR	25.2^a^	4.7	21.2^b^	5.4	19.5^c^	6.2	62.9**	0.43
QoL	62.4^a^	15.7	71.7^b^	18.1	75.9^c^	17.5	60.1**	0.42
Psychological flexibility	66.7^a^	14.3	79.0^b^	18.6	83.3^c^	19.4	54.3**	0.40
PHQ-9	10.2^a^	5.4	7.5^b^	5.6	6.5^b^	6.1	33.0**	0.29
GAD-7	10.0^a^	5.5	6.8^b^	5.4	6.1^b^	5.8	35.1**†	0.30

Means with different superscripts are significantly different for within participant comparisons at the level of *p* < 0.05

Significance of *F* test **p < 0.0001

^†^ indicates altered degrees of freedom 1.6, 136.1 with Greenhouse–Geisser correction

Effect size for partial eta squared (*η*_p_^2^) can be described as the following: small = 0.01–0.05, medium = 0.06–0.13, 0.14 = large =  ≥ 0.14

*FCR*, fear of cancer recurrence; *QoL*, quality of life; *PHQ-9*, Patient Health Questionnaire (9 items); *GAD-7*, Generalised Anxiety Disorder Assessment (7-items); *T1*, pre-intervention; *T2*, post-intervention; *T3*, 12 weeks following intervention

Analysis was carried out to assess if there was a significant reduction in FCR outcome score at T1, T2 and T3. The reduction to the mean scores on the FCR outcome measure from T1, T2 and T3 was statistically significant (*F*(2, 166) = 62.9, *p* < 0.001). Planned comparisons showed that FCR score significantly decreased from T1 to T2 (4.0 (95% CI, 2.7 to 5.2), *p* < 0.001) and from T1 to T3 (5.7 (95% CI, 4.3 to 7.1), *p* = 0.001) and finally from T2 to T3 (1.7 (95% CI, 0.5 to 2.9), *p* = 0.002).

The mean scores on the QoL outcome measure at T1, T2 and T3 also showed a statistically significant improvement (*F*(2, 166) = 60.1, *p* < 0.001). Planned comparisons showed that quality of life significantly increased from T1 to T2 (− 9.4 (95% CI, − 12.7 to − 6.0), *p* < 0.001), and from T1 to T3 (− 13.6 (95% CI, − 16.7 to − 10.4), *p* < 0.001), and finally from T2 to T3 (− 4.2 (95% CI, − 7.0 to − 1.4), *p* = 0.001). Table 3 shows the mean QoL score at each time point.

Data from two measures of psychological distress (PHQ-9 and GAD-7) were analysed to determine if there was a statistically significant reduction following group participation. The means scores on the PHQ-9 at T1, T2 and T3 revealed a statistically significant decrease (*F*(2, 166) = 33.0, *p* < 0.001).

Planned comparisons showed that depression significantly decreased from T1 to T2 (2.7 (95% CI 1.7–3.8), *p* < 0.001), and from T1 to T3 (3.8 (95% CI 2.5–5.0), *p* < 0.001). There was no significant difference between PHQ-9 scores at T2 and T3 (1.0 (95% CI − 0.1 to 2.2), *p* = 0.1).

The mean scores on the GAD-7 at T1, T2 and T3, with a Greenhouse–Geisser correction, revealed a statistically significant decrease (*F*(1.6, 136.1) = 35.1, *p* < 0.001).

Planned comparisons showed that generalised anxiety significantly decreased from T1 to T2 (3.1 (95% CI 2.1–4.2), *p* < 0.001), and from T1 to T3 (3.9 (95% CI 2.4–5.4), *p* < 0.001). There was no significant difference between GAD-7 scores at T2 and T3 (0.8 (95% CI − 0.3 to 1.8), *p* = 0.25).

The mean psychological flexibility scores at T1, T2 and T3 also showed a statistically significant increase (*F*(2, 166) = 54.3, *p* < 0.001). Planned comparisons showed that psychologically flexibility significantly increased from T1 to T2 (− 12.2 (95% CI − 16.3 to − 8.2), *p* < 0.001), and from T1 to T3 (− 16.6 (95% CI − 21.1 to − 12.1), *p* < 0.001), and from T2 to T3 (− 4.4 (95% CI − 7.9 to − 0.8), *p* = 0.01).

## Discussion

FCR persists over a number of years following treatment, if left untreated [28, 29]. It is currently unclear which psychological interventions are effective in supporting someone living with FCR in routine healthcare settings, particularly in usual care. The results of this evaluation demonstrate that a 6-week ACT-based group programme may be effective in improving scores on a range of outcome measures for people who have completed treatment for breast cancer. Results within the population evaluated show a reduction in FCR, increase in overall QoL, increased psychological flexibility, and decreased psychological distress. FCR, QoL and psychological flexibility changes further improved 3 months following group participation. The reduction in psychological distress persisted 3 months later. As it is not an aim of an ACT-based intervention to get rid of negative feelings and experiences, it may be the positive impact of the other outcomes which has led to the decline in participant experiences of depression and generalised anxiety within this population. Furthermore, the programme was found to be acceptable for this population, with a high engagement rate.

This service evaluation of a real-world NHS-embedded healthcare programme encompassed a population with a high initial level of FCR, with an average score of 25.2 on the FCRI-SF, compared to other studies involving breast cancer survivors [30]. There is some controversy over the optimal cut-off point for a clinically meaningful FCR score on this tool, with systematic review of studies encompassing 14,092 people affected by cancer, during or post-treatment, revealing a range of recommended cut-points between 13 and 22 [31]. This systematic review suggested optimal cut-point may be dependent on cancer diagnosis and treatment stage, with breast cancer survivors (*n* = 1540) found to have the third highest average FCRI-SF score (mean = 16.4).

It is not conclusive whether this group programme has reduced FCR scores to below clinically significant levels. We speculate there could be a number of reasons for the high FCR score pre-programme. Participation in the programme was voluntary; thus, those who decided to proceed may be more motivated due to high levels of FCR. Also, due to the well documented issue of FCR being an unmet need within cancer survivor populations [32], it may be that referrers within this service evaluation were only identifying those with higher levels of self-reported distress.

This service evaluation gives insight into the application of an ACT-based programme facilitated within hospital and community settings. The high retention rate of 89% found within this service evaluation is comparable to other published findings in similar format interventions for FCR (67% and 94.5%) [17, 18]. The uptake rate of 30% was low within this evaluation and this could be improved through appropriate education of referrers regarding to whom the group programme would be best suited. Formal use of screening tools by referring clinicians may also improve the overall uptake rate.

This programme was shown to meet the aims of an ACT-based intervention by improving participant psychological flexibility. Increased psychological flexibility has been attributed as a predictor of overall psychological wellbeing [33] and a small, observational study has revealed association between higher levels of psychological flexibility with better quality of life and lower psychological cancer-related stress [34]. The significant increase in psychological flexibility within this evaluation may suggest that participants experienced an improved ability to be open and aware of unwanted thoughts, feelings and experiences, whilst continuing to act according to deeply held values [35]. The continued significant increase in psychological flexibility beyond the completion of the programme may suggest that participants continue to utilise the techniques and tools learned beyond their participation in the programme.

The group programme benefited from several strengths. It was developed by experienced NHS staff as well as having further supervisory input from a clinical psychologist with expertise in ACT. The educational component delivered week 2 of the programme was developed by a Specialist Therapeutic Radiographer with 18 years qualified experience of working with cancer patients within NHS settings. Furthermore, there was a high return rate of outcome measures by those who completed the programme (87%). The collection of outcome measures at 12-week follow-up suggests that positive changes in FCR and QoL were not wholly attributable to an immediate effect of the routine weekly interaction with group participants and facilitators. The real-world nature of the results presented within this evaluation provides promising proof of concept data whilst acknowledging the limitations and ungeneralisable nature of the results.

The generalisability of this evaluation is limited by its sample size, restricted cancer type and having no control comparator group. It is acknowledged that the high level of FCR found within this sample is not generalisable to breast cancer survivor populations, overall. Referral to this group programme was predominantly based on clinical judgement during routine appointments. This is a subjective method, open to bias and reliant on patient vocalisation of FCR. Furthermore, participation in this programme was entirely voluntary and the participants were a potentially highly motivated, self-selecting sample of this population. Conclusions from these results are limited by a homogenous sample of all white, female, breast cancer survivors living in the West of Scotland and cannot be generalised to other ethnicities, geographical locations or cancer types.

These findings support the potential value in exploring the utilisation of an ACT-based intervention for survivors of a range of cancer types. It may also be of value to carry out an extended longitudinal study to determine if the scores continue to decrease or maintain beyond the 12-week follow-up evaluation. Future evaluation may wish to explore the comparison of facilitation of the groups by specialist staff, as in this programme, to less-costly facilitation by non-specialist facilitators. There is a lack of empirical research within this area [36] although there is evidence to suggest the efficacy of delivery of ACT-based interventions by non-mental health professionals [37].

## Conclusion

The evaluated ACT-based group programme provided a new healthcare intervention to those experiencing high levels of distress relating to FCR, following completion of active treatment for breast cancer, within an NHS health board in Scotland. The programme was found to be effective in reducing FCR whilst increasing QoL and psychological flexibility scores. These scores were shown to improve further between point of programme completion and at follow-up, 12 weeks later. Group participants also experienced a decrease in anxiety and depression. This service evaluation found overall high engagement levels for those who started the group programme. These results add to data evidencing efficacy of ACT-based treatment of FCR in routine service settings. RCT evaluation comparing this programme to other psychological and mindfulness-based interventions, as well as usual clinical care, is required to investigate efficacy compared to conditions that control for generic effects such as expectancy and therapist attention.

## Data Availability

All data was routinely collected, and healthcare data belonging to the NHS health board offering this group programme and as such are not publicly available.
